# The Mediating Role of Problematic Use of Loot Boxes Between Internet Gaming Disorder and Online Gambling Disorder: Cross-Sectional Analytical Study

**DOI:** 10.2196/57304

**Published:** 2024-09-20

**Authors:** Joaquín González-Cabrera, Vanessa Caba-Machado, Adoración Díaz-López, Susana Jiménez-Murcia, Gemma Mestre-Bach, Juan M Machimbarrena

**Affiliations:** 1 Instituto de Transferencia e Investigación (ITEI), Universidad Internacional de La Rioja (UNIR), Logroño, Spain; 2 Clinical Psychology Unit, Bellvitge University Hospital, Barcelona, Spain; 3 Psychoneurobiology of Eating and Addictive Behaviors Group, Bellvitge Biomedical Research Institute (IDIBELL), Barcelona, Spain; 4 CIBER de Fisiopatología de la Obesidad y Nutrición (CIBERobn), Instituto de Salud Carlos III, Madrid, Spain; 5 Department of Clinical Sciences, School of Medicine and Health Sciences, University of Barcelona, Barcelona, Spain; 6 Faculty of Psychology, Euskal Herriko Unibertsitatea (UPV/EHU), Donostia, Spain

**Keywords:** loot boxes, loot box, gaming, gambling, problematic, video games, game, games, addict, addiction, addictions, addictive, internet, virtual object, virtual objects, gamification, IGD, OGD, monetize, monetization, reward, rewards, incentive, incentives, internet gaming disorder, online gambling disorder

## Abstract

**Background:**

The video game industry has introduced a new form of monetization through microtransactions. A controversial example has been the so-called “loot boxes” (LBs) as virtual objects, which are randomized and bought with legal money. In recent years, LBs have come to connect 2 distinct problem behaviors, namely internet gaming disorder (IGD) and online gambling disorder (OGD). Many association studies have been conducted on the 3 constructs, but few have delved into the relationship of problematic use of LBs (PU-LB) with IGD and OGD.

**Objective:**

This study aims to explore the mediating role of the PU-LB between IGD and OGD.

**Methods:**

This cross-sectional and analytical study used incidental sampling in 24 Spanish schools. The final sample consisted of 542 participants (male: n=523, 96.5%; age: range 11‐30 y) who played video games, bought LBs, and had gambled online in the last 12 months. Participants then completed the Spanish versions of the Internet Gaming Disorder Scale–Short Form, Online Gambling Disorder Questionnaire, and PU-LB scale.

**Results:**

IGD scores were found to be significantly associated with both PU-LB (*r*=0.473, *P*<.001) and OGD (*r*=0.209, *P*<.001). Moreover, PU-LB was significantly associated with OGD (*r*=0.351, *P*<.001). The structural equation model results indicated that IGD had no significant direct effect on OGD (*P*=.903). However, the indirect effect of IGD on OGD through PU-LB was significant (*P*<.001). Therefore, PU-LB fully mediated the relationship between IGD and OGD. Furthermore, these results were found in the subsamples of both minors (<18 y) and young adults (≥18 y).

**Conclusions:**

It is suggested that there is a mediation effect of problematic LB use between internet gambling and online gambling problems in both minors and young adults. This has potential practical implications by providing more evidence on how LBs have become a hinge feature between 2 clinically relevant and independent issues. In this regard, adequate industry self-regulation is needed, and effective legislation for the protection of minors is necessary.

## Introduction

Video games are a form of interactive entertainment that has gained enormous popularity around the world. Video games can be seen even as a form of cultural expression because they reflect the creativity, values, beliefs, and experiences of both game developers and gamers alike [1].

According to a report by DFC Intelligence, 40% of the global population plays video games, which represents an estimated 3.1 billion people playing video games as of 2022 [2]. In 2022, the global video game industry reached a market size of approximately US $246 billion [3]. According to the Spanish Video Game Association [4], in 2022, a total of 18.2 million people in Spain were recorded as gamers (there will be more than 48 million inhabitants in 2023), with 53% being male and 47% female. According to the report, Spaniards play video games for 7.42 hours per week (8% less than in the previous report), and turnover has risen to 2012 million euros (an increase of 12% compared with the previous year). Furthermore, the age profile of Spanish video game players is mainly young, with 79% between 6 and 11 years old, 84% between 11 and 14 years old, and 71% between 15 and 24 years old [4]. In addition to the increase in video game sales, one of the factors behind this economic success lies in the incorporation of in-game purchases, which represent an increasingly large revenue stream for the industry [5,6]. In this regard, in 2023, a national study in Spain showed that 17.7% of adolescents between 14 and 18 years of age had gambled online. Of these, more than 50% claim to have gambled in the context of video games [7].

There is an ever-increasing number of video games that offer microtransactions (ie, the payment of a stipulated price for a specific, well-known skin or perk) [8]. Each video game features its own type of microtransactions, some of which are only of an aesthetic nature, while others may influence the dynamics of the game. Within the microtransactions, there is a special modality that in recent years has attracted the attention of researchers for its possible relation to random reward mechanisms: loot boxes (LBs), which are also called crates, cases, or chests. The acquisition of LBs involves the purchase of a virtual object (which can be acquired in various manifestations such as boxes, slot machines, chests, or in the form of animals), which is randomized and paid for with legal tender (this can be obtained from a prepaid card, a credit card payment or by prepurchasing currency from a game or environment to buy LBs) [9]. The fact that this virtual object is the product of a random reward is what has made it similar to gambling, as both share a random reward mechanism [9-11]. Paradigmatic examples of LBs in video games may be the case analyzed by Lemmens [12] on FIFA Ultimate Team and those analyzed by Xiao et al [13], with some of the top downloaded games for Android, such as Game of Thrones: Conquest or Pokemon GO, including LBs.

On the one hand, some primary studies have associated LB purchases with clinical problems with video games (usually assessed with questionnaires that follow the *Diagnostic and Statistical Manual of Mental Disorders, Fifth Edition* [*DSM-5*] criteria for internet gaming disorder [IGD]). Supporting evidence for this link has been found by authors in cross-sectional studies [6,14,15], but there is hardly any longitudinal evidence [16]. On the other hand, there is more evidence of a direct relationship between the purchase of LBs and problem gambling [5,10,11,15,17-24]. However, only a few studies have related the purchase of LBs to clinical problems of online gambling (following the *DSM-5* [25] and *International Classification of Diseases, 11th Revision* [*ICD-11*] criteria) [26], and almost all these have been cross-sectional studies. One of them is the research with minors and adults by González-Cabrera et al [27] carried out in Spain with a large sample of more than 6500 participants, where 3 out of every 10 participants purchased LBs in the last 12 months. Although limited, there is also evidence of the association of these problems over time in minors, with over 50% of LB purchasers still buying after 6 months [16]. Studies have also already been conducted as adults linking LBs to gambling over time [28].

Overall, we still need to answer the possible hypotheses raised by Spicer et al [29] about LBs and gambling: either (1) users who gamble in other environments buy more LBs; (2) buyers of LBs are more likely to start gambling, through the “gateway effect”; or (3) there is a complex and dynamic relationship between both behaviors, where gambling is known to interact with other risky behaviors. In line with the latter suggestion, LBs have been related to gaming and gambling problems in minors and mostly adults [19,27,29,30]. Nevertheless, the possible mediating role of LBs between both clinical problems has been much less addressed. LBs have been a novel and relatively massive phenomenon over the past 5 years (approximately). This phenomenon has become the hinge that can bridge 2 clinical and nosological entities that appear separately in the diagnostic manuals [25] and that have not been modified in the current revision of the *DSM-5-TR* [31]. As such, a pioneering study was conducted with adults in which the mediation of microtransaction engagement between gaming and gambling was analyzed [32]. This mediating construct was assessed using the Risky Loot-Box Index (RLI) [17], which captures cognitive concern about LB use, impulsive use, and chasing losses, but it has limitations in terms of validity and reliability. In addition, this study did not use a clinical assessment tool for online gambling (and did not consider research with minors).Despite these limitations, the results were interesting as they did not achieve a complete mediation (ie, the direct relationship between video game problems and betting was also significant). Overall, the results indicate that participants with IGD were more likely to purchase microtransactions and to report more gambling-related problems. It is also possible that there have been significant changes in the consumption of LBs since the 2020 release (King et al [32]), as this business model has become increasingly common and has grown in recent years [13].

This study, based on the study by King et al [32], included 2 clinical measures of online gaming and gambling problems and an instrument with adequate validity indicators of problematic LB use. In addition, a large sample of adults and minors (often less addressed in the literature) was included, with the latter requiring special safeguards regarding the LB phenomenon as covert gambling [9]. The authors posit the hypothesis that there is no direct relationship between clinical problems with video games and online gambling, unless there is a problematic use of LBs (PU-LB) that mediates this relationship and therefore generates a significant indirect effect between the 2 clinical variables in the model. Thus, the aim was to perform a mediation of the PU-LB scale between IGD and online gambling disorder (OGD) in a sample of minors and young adults.

## Methods

### Design and Recruitment

This study used a cross-sectional design. The sampling was incidental and was carried out in 24 nonuniversity educational centers in 8 Spanish regions (ie, Asturias, Cantabria, Castilla la Mancha, Castilla y Leon, Comunidad de Madrid, Comunidad Foral de Navarra, Comunidad Valenciana, and País Vasco). The educational stages covered ranged from compulsory secondary education (approximately between 11 and 16 y of age) to baccalaureate education (approximately between 16 and 18 y of age), with the addition of vocational training (where the ages range between 15 and 30 y old). This sample is part of a larger study on internet risks in adolescence. The final sample consisted of participants (both younger and older than 18 years) who had answered “yes” to the following 3 questions: have you played video games in the last 12 months, have you bought any video game LBs with money in the last 12 months, and have you gambled online in any type of game in the last 12 months?

### Assessment Instruments

Participants were initially asked sociodemographic questions (gender, age, study center, and province).

IGD was assessed with the Spanish version of the Internet Gaming Disorder Scale–Short Form (IGD9-SF) [33-35]. This scale consists of 9 items based on the *DSM-5* criteria for IGD (eg, “Have you deceived any of your family, therapists, or friends about the time you spend gaming?”) [25]. The scale response options range from 0 (never) to 4 (very often). The total score ranges between 0 and 36, with greater scores suggesting higher symptom-severity of disordered gaming. In terms of internal reliability, the Cronbach α coefficient and ω coefficient in the present sample were 0.85 and 0.86, respectively.

As for OGD, this construct was evaluated with the Online Gambling Disorder Questionnaire (OGD-Q) [36]. This scale consists of 11 items that assess OGD in adolescence (eg, “Do you feel nervous, irritated, or angry when trying to reduce or stop gambling online?”). The scale response options range from 0 (never) to 4 (every day), with greater scores suggesting higher symptom-severity of disordered gambling. The total score ranges between 0 and 45. The scale Cronbach α coefficient and ω coefficient were 0.92 and 0.92, respectively.

The PU-LB scale [27], consists of 18 items assessing the potentially problematic nature of engaging in LB purchasing behavior (eg, “Loot boxes have caused problems in my life (either social, economic, family, school, or work. etc” or “I usually buy loot boxes to feel better or happier”). The scale response options range from 0 (strongly disagree) to 5 (strongly agree), with total scores ranging from 0 to 90, where higher scores suggest a more PU-LB. In relation to its internal reliability, the Cronbach α coefficient and ω coefficient in the present sample were 0.92 and 0.87, respectively.

### Procedure

The survey was conducted online through the Survey Monkey platform on either a mobile device or computer. The participants were given access to and supervised by their teachers. The evaluation was carried out in educational centers (during the school timetable). The researchers previously trained the teachers in data collection. In addition, participant detection mechanisms were enabled, such as those suggested by Niessen et al [37], which included maximum response time “longstring” and “person-fit statistics.” The time needed to complete the questionnaires ranged between 5 and 15 minutes, depending on students’ age and reading comprehension.

### Ethical Considerations

The study was conducted with the authorization of all the participants in the investigation and with the consent of the school directors, students, and families. Students and families’ collaboration was voluntary, anonymous, and disinterested. The project was approved by the Research Ethics Committee of International University of La Rioja (Spain) (PI007-2020 y PI001/2021), and the Juvenile Prosecutor’s Office was informed. The study received consent from all participants and school principals. Consent forms were sent to parents or guardians of participants younger than 18 years, and the purpose of the study was explained. About 0.8% of the participants did not want to respond to the questionnaire, while less than 1% of parents or guardians refused participation. Participants older than 18 years provided informed consent when completing the survey. Although there were no formal exclusion criteria, except for refusal to participate by parents or guardians for the overall sample, to be included in this study, participants had to answer affirmatively to a dichotomous question (yes or no) on whether they had played video games in the last 12 months, whether they had gambled online in the last 12 months, and whether they had bought LBs in the last 12 months. Only those who answered “yes” to each of these questions were assessed and included in the study.

### Statistical Analysis

The SPSS (version 26; IBM Corp) program was used to (1) explore and screen all data through descriptive statistics; (2) test for reliability by Cronbach α, ω coefficient, and normality through skewness and kurtosis; and (3) explore the relationships between variables through bivariate correlations. The absolute values of skewness and kurtosis are normal when they are below ±3 for skewness and ±10 for kurtosis [38].

MPLUS (version 8.0; Muthen & Muthen) [39] was used to test (1) the factor structure of the PU-LB through confirmatory factor analysis (CFA) and (2) the relationships between IGD, PU-LB, and OGD through structural equation modeling analysis. The Maximum Likelihood Robust Estimator was used, and the fit of the model was estimated with the most reliable fit indices [40]: the Satorra-Bentler chi-square (S-B*χ*^2^), the root mean square error of approximation (RMSEA), the comparative fit index (CFI), the Tucker-Lewis index (TLI), and the standardized root mean square residual (SRMR). A model was considered to adequately fit the data at values ≥.90 for the CFI and TLI, with values above .95 preferred, and values ≤.08 for the RMSEA and SRMR [38]. The significance of mediational paths was tested by means of bias-corrected bootstrapping with 5000 samples.

## Results

### Participants, Descriptive Statistics, Normality, and Reliability

The sample was composed of 542 participants (male: n=523, 96.5%) who played video games, purchased LBs, and gambled online in the last 12 months. The average age of the sample was 17.78 (SD 2.78) years (age: range 11‐30 y), out of which 47.2% (n=256) were minors (mean_age_ 15.3, SD 1.71 y) and the remaining 56.3% (n=306) were young adults older than 18 years (mean_age_ 19.6, SD 1.88 y). There were also 107 (19.7%) students in compulsory secondary education, 36 (6.6%) in baccalaureate education, and 399 (73.7%) in vocational training.

Descriptives, segregated by age groups (minors and adults), for all variables, including the means, SDs, and Pearson bivariate correlations between the variables of the study, are presented in Table 1. Moreover, for the total sample (minors and adults), the descriptive statistics were as follows: for IGD, mean 8.216 (SD 0.301), skewness=1.023, and kurtosis=0.858; for PU-LB, mean 12.583 (SD 14.890), skewness=1.590, and kurtosis=2.794; and for OGD, mean 4.053 (SD 7.104), skewness=2.585, and kurtosis=7.385.

IGD scores were found to be significantly associated with both PU-LB (*r*=0.473, *P*<.001) and OGD (*r*=0.209, *P*<.001). Additionally, PU-LB was significantly associated with OGD (*r*=0.351, *P*<.001).

**Table 1.** Correlation matrix, descriptive statistics for internet gaming disorder (IGD), problematic use of loot boxes (PU-LB), and online gambling disorder (OGD). The results for the minors (n=236) are shown below the diagonal. The results for adults (n=306) are shown above the diagonal.

	Variables			Minors (n=256)				Young adults (n=306)			
	IGD	PU-LB	OGD	Questionnaire Score, mean (SD)		Skew^a^	Kurt^b^	Questionnaire Score, mean (SD)		Skew	Kurt
IGD	—^c^	0.477^d^	0.229^d^	8.22 (7.07)		1.02	1.23	8.2 (7.48)		1.025	0.64
PU-LB	0.484^d^	—	0.354^d^	15.33 (14.83)		0.96	0.12	10.46 (14.6)		2.203	6.02
OGD	0.179^d^	0.410^d^	—	3.3 (6.09)		3.19	12.18	4.62 (7.75)		2.282	5.33

a Skew: skewness.

b Kurt: kurtosis.

c Not applicable.

d All correlations were significant at *P*<.001.

### Structural Mediation Model

Before testing the structural mediation model, the CFA model of PU-LB (which had been validated through exploratory factor analysis but not through CFA) yielded some evidence for satisfactory fit (S-B*χ*^2^_125_=300.207; RMSEA=.051, 90% CI .044-.058; CFI=.929; TLI=.913; SRMR=.054). The IGDS9-SF and OGD-Q had been previously validated and had shown good structural properties in previous studies [33,36].

**Figure 1.**
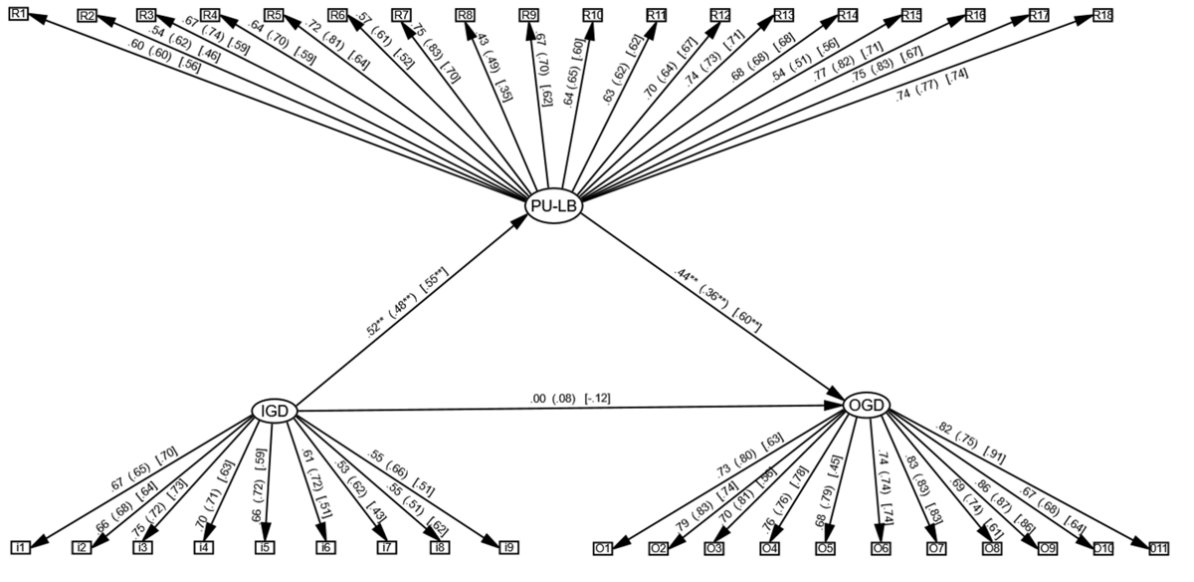
Structural equation model for IGD, PU-LB, and OGD with standardized factorial loadings. **P<.001; the values provided are in the format “total sample, (young adults), [minors].” IGD: internet gaming disorder; PU-LB: problematic use of loot boxes; OGD: online gambling disorder.

Figure 1 displays standardized path coefficients for the structural equation modeling with the total sample and subsamples of minors and young adults. The model for the overall sample had an adequate fit (S-B*χ*^2^_647_=1156.821; RMSEA=.038, 90% CI .035-.042; CFI=.924; TLI=.918; SRMR=.055). All items loading onto latent variable were significant (*P*<.001) and ranged from .45 to .86. Moreover, model for the subsample of young adults (*n*=306) (S-B*χ*^2^_647_=1057.634; RMSEA=.046, 90% CI .041-.050; CFI=.910; TLI=.908; SRMR=.064), and minors (n=236) (S-B*χ*^2^_647_=980.688; RMSEA=.047, 90% CI .041-.052; CFI=.892; TLI=.882; SRMR=.065), showed inconsistent indexes for CFI and TLI, and while the RMSEA and SRMR values remain acceptable, the values for the CFI fail to meet the cut-off (≥.90). However, Raykov [41] defended that CFI is a measure based on noncentrality and therefore, could be biased.

The results in the total sample indicated that IGD did not have a significant direct effect on OGD (*β*=.004, *P*=.903). However, the indirect effect of IGD on OGD by PU-LB was significant: (*β*=.223, 95% CI .131-.338, *P*<.001). Therefore, PU-LB fully mediated the relationship between IGD and OGD. In addition, these results were also found in the subsamples of minors (*β*=.327, 95% CI .177-.561, *P*<.001) and young adults (*β*=.187, 95% CI .072-.324, *P*<.001).

## Discussion

The business model of many video game companies has been linked to LBs; this is worrying for many sectors of society, and this has generated wide interest in the academic and research context. Moreover, LBs appear to be a hinge that links 2 problem behaviors (IGD and OGD). In this regard, at an early stage in the study of LBs, King et al [32] conducted a mediation of microtransaction engagement between gaming and gambling in adults. There is evidence of an increase in the number of video games with LBs in recent years [13] and the difficulty for laws restricting the use of LBs to be effective [42]. The aim was of this study to perform a model that would allow us to analyze whether there would be a direct relationship between IGD and OGD in video game users who are LBs buyers and online gamblers in the last year or if, on the contrary, there would be an indirect effect thanks to the mediation of the PU-LB. The results suggest that because of the significant indirect effect (and the absence of direct effect), there is a total mediation effect. This reinforces the idea that the PU-LB may be a problem connecting 2 different problem behaviors (IGD and OGD) and that LBs, as a random reward mechanism, can be associated with gambling as a gateway or as a further gambling mechanism [29]. Thus, LBs seem to have spurred and connected 2 very pernicious issues to each other, adding a layer of complexity to the problem. Furthermore, this phenomenon is especially worrisome because minors are involved in these mechanisms (the model adjusts for the total sample and for minors and adults). From these data, important theoretical implications are derived. On the one hand, problems with gaming do not have a direct effect on gambling, and on the other hand, PU-LB generates a full mediation, justifying that hinge role. It should be noted that the entire study sample consisted of buyers of LBs, so it is not only necessary to buy them but also to present a problem with them. This is also related to the emphasis of the *ICD-11* [23] on the consequences that behavioral disorders should have. With the above, it is clear that industry mechanisms do not seem to be sufficient to regulate this process [43], nor are the governmental measures taken in some countries to curb the problem [44].

The study by King et al [32], as well as other studies, used the RLI to assess the risk of LB use [10,28,32]. In general, the RLI shows deficiencies in its psychometric validation process, as well as drawbacks for not covering other key aspects related to the problems that may arise due to the behavior of purchasing LBs (eg, impulsivity to buy more LBs, personal consequences, salience of play time or guilt, among others). For this reason, we used the PU-LB [16], which presents adequate validity and reliability indicators; its content includes validity indicators of general problems about LBs and specific indicators regarding the association among LBs, gaming, and gambling (mood regulation through the purchase or opening of LBs, postponing activities to get LBs, feeling the urge to buy them, thinking about the purchase activity or feeling bad about the time or money invested, etc). Furthermore, contributing to the pioneering work of King et al [32] is the use of clinical questionnaires (IGDS9-SF and OGD-Q) based on the diagnostic criteria of the *DSM-5* [25] and *ICD-11* [45] (including minors and adults). In this sense, there are no data to compare with, as this study has a singular focus.

This study has some relevant limitations. First, only self-report measures were used, which may generate response bias and social desirability bias. Second, there may be a retrospective recall bias, as participants were asked to think back to what they did in the last 12 months. Third, although the sample of participants was large and geographically dispersed, the sampling was not random, so it is not representative of the Spanish context. Fourth, there is an overrepresentation of male participants in the study, which is a common issue in many studies, since consumers of video games, gambling and LBs are mostly male. However, these data also indicate that boys require special attention regarding the LB problem (at least in the Spanish context). Fifth, the OGD-Q questionnaire is validated in Spanish adolescents (up to the age of 19 years) and not in adults. Although the reliability indicators are adequate, this may be a limitation of the study. Sixth, all parameters, apart from the OGD variable in the group of minors, exhibited skewness and kurtosis values indicative of a normal distribution. However, it is emphasized that the lack of normality does not pose a methodological obstacle, as a robust approach was used to effectively address the presence of nonnormal distributions in the statistical analysis. Finally, the fit indices of the model for minors are slightly below the thresholds considered good (particularly the CFI and TLI). However, this may be due to the nature of the constructs and the fact that online gambling is an illegal activity for minors; therefore, the data related to the OGD may have affected the model in general.

Given these potential limitations, future research should include longitudinal designs that take into account the variables used in this study and answer the questions posed by Spicer et al [29] on the “gateway effect” of LBs or the relationship between who opens and who purchases LBs, as opening is likely to focus more on gaming problems and purchasing is likely to focus more on gambling. As such, exploring the independent and additive effect of both roles may be an area for future research. However, this study has been able to address questions also raised by these authors in relation to the relationship between gaming and gambling problems. This study has potential practical implications by providing more evidence on how LBs have become a hinge feature between 2 clinically relevant and independent issues. While there has been very strict legislation on LBs in countries such as Belgium [42], it has not been effective because it has not been properly enforced. Despite this situation, there is still a need for politicians to properly regulate the LB framework and prevent minors from purchasing LBs. The key aspect is to enforce the law and provide resources to do so. In the Spanish context, there has been a draft bill in place since 2022 to regulate random reward mechanisms associated with interactive software products [46]. This draft bill has basic limitations such as a remarkably restrictive definition of a LB (article 3, point C), which, if approved, will be clearly insufficient. This is why, in the Spanish case, it is necessary to improve legislative efforts and add other actions. In this sense, it could help to make it a requirement for the video game industry to include information on LBs (within the framework of the Pan-European Gaming Information [PEGI] system), but this must be clear, specific, and comprehensible [43]. Finally, and perhaps most importantly, there is also a need for psychoeducational actions aimed primarily at preventing the purchase of LBs by minors. These actions should be evidence-based. Education of minors and their families is key and is the future course of action.

In conclusion, this study suggests that there is a mediation effect of problematic LB use between IGD and OGD in both minors and young adults.

## Abbreviations

CFA: confirmatory factor analysis
CFI: comparative fit index
*DSM-5*: *Diagnostic and Statistical Manual of Mental Disorders, Fifth Edition*
*ICD-11*: *International Classification of Diseases, 11th Revision*
IGD: internet gaming disorder
IGD9-SF: Internet Gaming Disorder Scale–Short Form
LB: loot box
OGD: online gambling disorder
OGD-Q: Online Gambling Disorder Questionnaire
PEGI: Pan-European Gaming Information
PU-LB: problematic use of loot boxes
RLI: Risky Loot-Box Index
RMSEA: root mean square error of approximation
S-B*χ*^2^: Satorra-Bentler chi-square
SRMR: standardized root mean square residual
TLI: Tucker-Lewis index
